# Generation of Antibodies against Foot-and-Mouth-Disease Virus Capsid Protein VP4 Using Hepatitis B Core VLPs as a Scaffold

**DOI:** 10.3390/life11040338

**Published:** 2021-04-11

**Authors:** Jessica Swanson, Rennos Fragkoudis, Philippa C. Hawes, Joseph Newman, Alison Burman, Anusha Panjwani, Nicola J. Stonehouse, Tobias J. Tuthill

**Affiliations:** 1 The Pirbright Institute, Pirbright GU24 0NF, UK; j.j.swanson@leeds.ac.uk (J.S.); r.fragkoudis@ed.ac.uk (R.F.); pippa.hawes@pirbright.ac.uk (P.C.H.); joseph.newman@pirbright.ac.uk (J.N.); alison.burman@pirbright.ac.uk (A.B.); panjwanianusha@googlemail.com (A.P.); 2 Faculty of Biological Sciences, University of Leeds, Leeds LS2 9JT, UK; n.j.stonehouse@leeds.ac.uk

**Keywords:** picornavirus, FMDV, VLP, capsid, antibodies

## Abstract

The picornavirus foot-and-mouth disease virus (FMDV) is the causative agent of the economically important disease of livestock, foot-and-mouth disease (FMD). VP4 is a highly conserved capsid protein, which is important during virus entry. Previous published work has shown that antibodies targeting the N-terminus of VP4 of the picornavirus human rhinovirus are broadly neutralising. In addition, previous studies showed that immunisation with the N-terminal 20 amino acids of enterovirus A71 VP4 displayed on the hepatitis B core (HBc) virus-like particles (VLP) can induce cross-genotype neutralisation. To investigate if a similar neutralising response against FMDV VP4 could be generated, HBc VLPs displaying the N-terminus of FMDV VP4 were designed. The N-terminal 15 amino acids of FMDV VP4 was inserted into the major immunodominant region. HBc VLPs were also decorated with peptides of the N-terminus of FMDV VP4 attached using a HBc-spike binding tag. Both types of VLPs were used to immunise mice and the resulting serum was investigated for VP4-specific antibodies. The VLP with VP4 inserted into the spike, induced VP4-specific antibodies, however the VLPs with peptides attached to the spikes did not. The VP4-specific antibodies could recognise native FMDV, but virus neutralisation was not demonstrated. This work shows that the HBc VLP presents a useful tool for the presentation of FMDV capsid epitopes.

## 1. Introduction

Foot-and-mouth disease virus (FMDV) is the causative agent of foot-and-mouth disease, an economically important disease of livestock such as cattle and pigs [1]. In countries normally FMDV-free an outbreak can incur huge costs, with the 2001 FMDV outbreak in the UK estimated to have cost the agriculture industry alone GBP 3.1 billion [2]. FMDV is also endemic in large regions of the African and Asian continents, contributing to poverty and poor nutrition in rural communities and limiting trade opportunities for these countries [3].

There are seven distinct serotypes of FMDV (A, C, O, Asia1, SAT 1 (Southern African Territories 1), SAT 2 and SAT 3) and there are high levels of genetic variation within each serotype [1]. Serotype A is considered the most diverse and contains three geographically distinct topotypes and across these there are 26 distinct genotypes [4,5]. This creates a problem for vaccination programs, as there is little cross-protection from the vaccine between different genotypes of the same serotype.

FMDV is classified in the *Aphthovirus* genus, within the *Picornaviridae* family. Additional well-studied viruses in the family include those in the *Enterovirus* genus, such as poliovirus (PV) and human rhinovirus (HRV). Picornaviruses have a pseudo T = 3 (pT = 3) icosahedral non-enveloped capsid and are composed of four viral proteins. VP1, VP2 and VP3 form the outer layer of the capsid, while VP4 is thought to be internal [6]. VP4 is a small hydrophobic protein which is thought to be transiently exposed at the capsid surface in a process known as capsid "breathing" and is externalised to play an important role during cell entry [7,8,9,10,11].

Previous work in several members of the *Enterovirus* genus has shown that antibodies against VP4 can neutralise virus infection. Initially, it was discovered in PV that antibodies raised against a synthetic VP4 peptide could neutralise PV [12]. Further studies have shown that antibodies against the N-terminus of HRV VP4 are also able to neutralise virus infectivity [13,14]. Antisera raised against the N-terminal 30 amino acids of HRV14 VP4 were shown to be able to neutralise other serotypes of HRV, HRV16 and HRV29, indicating that the N-terminus of HRV14 VP4 elicits a cross-serotypic neutralising response [13]. In FMDV, VP4 is very highly conserved across all serotypes, suggesting that this may also present an interesting novel target for antibodies generated against FMDV.

Recently, the N-terminal 20 amino acids of enterovirus-A71 (EVA71) VP4 were displayed at the major immunodominant region (MIR) of the well-established hepatitis B core (HBc) virus-like particles (VLP) [15]. The serum from mice immunised with the VP4N20-displaying VLP was able to neutralise different genotypes of EVA71 in vitro and neutralise virus and protect neonatal mice from infection [15]. This study showed that protective responses to picornavirus VP4 can be induced by displaying VP4 epitopes on a VLP.

**Figure 1:**
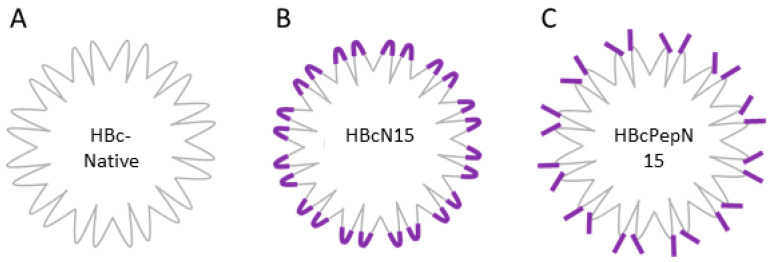
Schematic of HBcAg presentation methods. (**A**) HBc-native VLP, (**B**) HBcN15: HBc VLP with the N-terminal 15 amino acids of FMDV VP4 (purple) inserted, allowing this sequence to be presented on the major immunodominant region. (**C**) HBcPepN15: HBc-native VLP decorated with VP4N15 peptides via a peptide tag with affinity for the HBc spike.

The HBc is a self-assembling VLP derived from the HBcAg protein of the hepatitis B virus. Producing recombinant HBc, VLPs displaying an epitope of choice can be achieved through cloning the sequence of the antigen into one of three locations on the HBc VLP. The possible insertion sites are at either terminus or in the major immunodominant region (MIR). The most common insertion site is the MIR at the top of the spike of the HBcAg protein and this is tolerant of the largest insertions (Figure 1). The HBc VLP forms two types of capsids, containing either 240 or 180 copies of the HBcAg protein which form capsids of either 34 nm or 30 nm in diameter with triangulation numbers of T = 4 and T = 3, respectively [16].

Previous work has shown that the sequence of the 238 amino acid GFP has been successfully inserted into the VLP at the MIR [17]. Several pathogen antigens have also been displayed on the HBc using this approach, including the FMDV VP1 protein and a region of the circumsporozoite protein from Plasmodium falciparum, a causative agent of malaria [18,19].

An alternative approach is to attach exogenous peptides to the top of the spike region and there are two approaches to this previously described in the literature. Firstly, peptides can be attached at random by chemical cross-linking. This has been described as a successful approach for presenting peptides, but it is not possible to predict the orientation of the peptides on the VLP [20]. Secondly, it is possible to utilise an interaction between the HBcAg protein and a short peptide sequence [21]. This interaction relies on opposing charges between the peptide and the top of the spike, and this protein–protein interaction holds the peptide antigen onto the VLP. Previous work has shown that this sequence can be used as a tag to attach peptides containing the tag onto the MIR of the HBc VLP. This has successfully allowed for the display of an influenza A M2e peptide on the HBc VLP and the use of this antigen to induce an M2-specific virus neutralising immune response [22].

The study presented here compared two different approaches to display antigens on the HBc VLP, a recombinant VLP with a FMDV VP4 sequence displayed by insertion in the MIR (termed HBcN15), and a VLP decorated with a FMDV VP4 peptide attached by the specific peptide sequence (termed HBcPepN15) (schematic of approaches is shown in Figure 1). We used a 15 amino acid sequence as the peptide epitope, which is shorter than used in previously reported studies. This part of VP4 has been shown to be transiently exposed at the surface of the HRV capsid ([23] and reviewed in [24]). We hypothesised that this most N-terminal sequence would be the part of FMDV VP4 most likely to be exposed and that it would, therefore, be beneficial to focus the immune response towards this.

The antibody response in mice to both presentation methods were compared and the effect of VP4N15-specific antibodies on FMDV was investigated.

## 2. Materials and Methods

### 2.1. Construction of HBc Expression Vectors and HBc VLPs

The plasmid pCoHBc190 C61A encoded the hepatitis B core (HBc) protein and contained *EcoR*I and *Eag*I restriction sites to allow for the insertion of sequences that would be displayed at the major immunodominant region (MIR) at the top of the HBc spike [25]. This plasmid was kindly provided by Dr Sam Stephen at the University of Leeds and was used to produce a HBc VLP (without insertion at the MIR), called HBc-C61A. 

A synthetic DNA sequence encoding the N-terminal 15 amino acids of VP4 (GAGQSSPATGSQNQS) from FMDV serotype O was produced by GeneArt (Thermo Fisher Scientific, Waltham, MA, USA) and inserted into the cloning site at the MIR using *EcoR*I and *Eag*I restriction sites in pCoHBc190 C61A to make the vector pHBcN15. The DNA sequence was GAATTCGGGGCTGGACAATCCAGTCCAGCGACCGGCTCGCAGAACCAATCTGCTAGC and the restriction sites used are underlined. This plasmid was used to produce a HBc VLP (displaying the 15 amino acids of VP4), called HBcN15. 

A synthetic DNA sequence was designed to remove the C61A mutation and the restriction sites at the MIR from pCoHBc190 C61A, thus returning the encoded protein to a "native" form. This was cloned into the pCoHBc190 C61A vector using *Nco*I and *EagI* restriction sites to create pHBc-native which was used to produce HBc VLPs called HBc-native.

### 2.2. Expression of HBcAg Proteins in E. coli

BL21 (DE3) *E. coli* (New England Biolabs) were transformed with the HBcAg expression vectors, pCoHBc190 C61A, pHBcN15 and pHBc-native. These were plated out onto Luria-Bertani (LB) plates, containing 50 µg/mL kanamycin. Overnight, bacterial cultures were grown from individual colonies. The following morning, 1 mL of overnight culture was used for every 100 mL of expression culture and incubated at 37 °C in a shaking incubator. The optical density (OD) at 600 nm (OD600) was monitored and expression was induced using 1 mM IPTG when the OD600 was between 0.6–0.8. The bacterial cultures were incubated for a further 3 h. Bacterial pellets were collected by centrifugation at 7000× *g* for 20 min.

### 2.3. Purification of HBcAg VLPs

Bacterial pellets were resuspended in lysis buffer (20 mM Hepes, 250 mM NaCl, pH 7.5) with protease inhibitor (cOmplete^TM^ mini EDTA-free, Roche Diagnostics Ltd, Burgess Hill, UK) and 2.5 units/mL of Benzonase nuclease (Merk Millipore, Burlington, MA, USA). The bacterial suspension was sonicated using two rounds of 10 × 30 s on 30 s off at an amplitude between 5 and 10. Insoluble debris was pelleted at 7000× *g* for 30 min. Soluble protein was precipitated with 40% (*w*/*v*) ammonium sulphate overnight and pelleted at 17,648× *g* in the JA-10 rotor. The protein pellet was resuspended in capsid buffer (20 mM Hepes, 250 mM NaCl, 2 mM DDT, pH 7.5) and layered onto a 20–60% (*w*/*v*) sucrose gradient. The gradient was centrifuged for 3 h at 151,000× *g* in the SW32Ti rotor at 4 °C. Peak fractions were identified by SDS-page and pooled for buffer exchange into PBS (Gibco Thermo Fisher Scientific, Waltham, MA, USA). The pooled peak fractions were purified through a second sucrose gradient to ensure purity.

The presence of purified VLPs was confirmed by Western blot using the anti-HBcAg monoclonal 10E11 (Abcam, Cambridge, UK). The secondary antibody used was horseradish peroxidase conjugated anti-mouse (Santa Cruz, Dallas, TX, USA).

### 2.4. Attachment of VP4N15 Peptide to HBc-Native VLPs

Peptides comprising the N-terminal 15 amino acids of FMDV VP4, followed by a lysine and histidine linker and with a tag to allow attachment to the HBc-native VLPs (as described previously [22]) were purchased from Peptide Synthetics. The peptide sequence was myr-**GAGQSSPATGSQNQS**KKKKKKHHHHHHGSLLGRMKGA. The peptide was modified with a myristate at the N-terminus (native VP4 is myristoylated) and the VP4 sequence is shown in bold and the spike tag is underlined. HBc-native VLPs were incubated with peptides at a 1:4 molar ratio for a minimum of 1 h shaking at room temperature. After incubation, the mix was diluted, and unbound peptide was removed using a 100 kDa MWCO Amicon centrifugal device (Merck Millipore, Burlington, MA, USA). The sample was washed three times with PBS (Gibco Thermo Fisher Scientific, Waltham, MA, USA) and the final concentration determined by Bradford assay.

### 2.5. Negative-Stain Transmission Electron Microscopy

Electron microscopy was used to confirm whether the purified HBc VLPs had self-assembled into particles with the expected appearance. Seven microlitres of purified sample were placed on glow discharged, Formvar/carbon coated copper TEM grids (Agar Scientific, Stansted, UK) and negatively stained for one minute using 2% aqueous uranyl acetate. The prepared grids were imaged at 100 kV in a FEI Tecnai 12 TEM with Tietz F214 CCD camera.

### 2.6. Immunisation of Mice with VLPs

Balb/c mice were used to study the immune response generated against the N-terminal 15 amino acids of VP4, displayed using two different HBc VLPs. Six female mice of between 5–6 weeks of age were immunized with 10 µg of VLP antigen mixed at a 1:1 ratio with Titermax adjuvant (TiterMax USA, Norcross, GA, USA) in a total volume of 250 µL. Three mice were immunized with a control VLP without VP4 epitope. Two boosts were carried out with 10 µg of antigen at 2 and 4 weeks after the initial immunization. Before immunization and, at days 10 and 24, blood samples were collected from the tail vein. At the end of the study, the mice were euthanised and the terminal bleed was collected via cardiac puncture. Animal experiments were approved by The Pirbright Institute Animal Welfare and Ethical Review Body and were performed under license (PPL number: 7008958) and in accordance with all relevant UK guidelines and regulations.

### 2.7. Analysis of Serum by Peptide and Sandwich ELISAs

Peptide ELISAs were used to evaluate the antibody response to the VLP immunisation. ELISA plates were coated with 1 µg/mL of HBc-native VLP or VP4N15 peptide overnight. After washing to remove unbound antigen, the plate was blocked with 5% fat free milk in PBS-T (PBS + 0.1% Tween 20). Mouse serum was diluted in 1% fat free milk in PBS-T and incubated on the plate at 37 °C with gentle mixing for 1 h. After washing, the HRP-conjugated secondary antibody (Dako Agilent, Santa Clara, CA, USA) was added in 1% fat-free milk in PBS-T and incubated at 37 °C with gentle mixing for 1 h. Sigmafast OPD was added at a final concentration of 0.4 mg/mL and incubated for 10 min. Once the colour had developed, the reaction was stopped with 1M sulphuric acid. The absorbance at 490 nm was measured using the EMax plate reader (Molecular Devices, San Jose, CA, USA).

Sandwich ELISAs were used to determine if the mouse serum recognised intact FMDV O1 Manisa. A 96 well plate was coated in 1 µg/mL of bovine integrin (kindly provided by Alison Burman) overnight [26]. After blocking, the FMDV O1 Manisa was diluted in 1% fat free milk in PBS-T and incubated on the plate at 37 °C with gentle mixing for 1 h. Primary and secondary antibodies and OPD were added as per the peptide ELISA and absorbance at 490 nm was measured using a Chameleon plate reader (Hidex, Turku, Finland).

### 2.8. Plaque Reduction Neutralisation Assay

Plaque reduction neutralisation assays were used to determine if the mouse serum neutralised FMDV, as follows. A volume of 1 µL of test serum, control neutralising serum or PBS were incubated with 12.5 µL of FMDV infected cell lysate for 1 h at 37 °C. After incubation a tenfold dilution series was made. Six well plates with BHKs at approximately 80% confluency were washed with sterile PBS. After the PBS was removed, 100 µL of each virus dilution was added to a well and incubated at 37 °C for 15 min. After incubation, 4 mL of Eagle’s overlay containing 0.6 g low melting point agarose (indubiose), 1% FBS, 5% tryptone phosphate broth, 100 units/ mL penicillin and 100 µg/ mL streptomycin was added on top of the cells. Once the overlay had set, the plaque assay was incubated at 37 °C for two days. After incubation, 4 mL of methylene blue stain (0.1% methylene blue, 3.2% formaldehyde in PBS) was added to fix and stain the cells. This was left overnight before being removed. The virus titre was calculated by counting the plaques in each dilution to establish plaque forming units per mL (PFU/mL).

## 3. Results

### 3.1. HBcAg VLPs Displaying VP4N15 Were Generated Using Two Methods of Antigen Presentation

**Figure 2:**
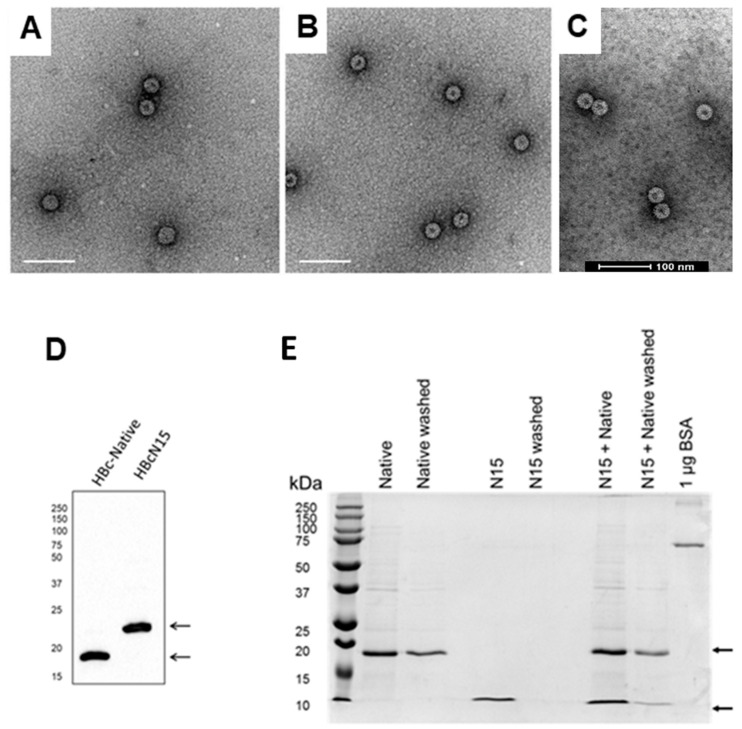
Formation of VP4N15 HBcAg VLPs (**A**) Purified HBc-C61A VLP, (**B**) HBcN15 VLP and (**C**) HBcPepN15 were visualised by negative stain TEM. The scale bars denote 100 nm. (**D**) HBc-Native and HBcN15 recombinant proteins were confirmed as having the expected molecular weights and reactivity by Western blot with anti-HBcAg antibody. The positions of molecular weight markers are shown to the left. Arrows on the right indicate the expected sizes of HBc protein. (**E**) HBc-native VLP only (native), VP4N15 peptide only (N15) or VLPs + VP4N15 peptide (N15 + native) before and after washing to remove free peptide. Samples were compared by Coomassie stained SDS-PAGE. The ladder and molecular weight are shown to the left. Arrows on the right indicate the expected sizes of HBc protein and the peptide (21 kDa and 4 kDa).

Recombinant HBc VLPs, which contained the N-terminal 15 amino acids of FMDV VP4 inserted within the MIR (termed HBcN15) and HBc VLPs without insertion at the MIR (termed HBc-C61A), were expressed in *E. coli* and purified by sedimentation through sucrose density gradients. The recombinant VLPs were visualised by negative stain TEM. Both types of particles had the expected size and appearance (Figure 2A,B), confirming that the insertion of VP4N15 had not visibly altered the overall structure compared to the HBc-C61A VLPs.

The HBcAg protein in HBc-native and HBcN15 VLPs was characterized by SDS-PAGE and Western blotting, which confirmed the expected sizes (Figure 2D).

HBc-native VLPs were incubated with excess VP4N15 peptide containing a spike-tag at the C-terminus. Unbound peptide was predicted to be removed through washing in a centrifugal concentrator device with a 100 kDa molecular weight cut off to allow the VLP and VLP-peptide complex to be retained but the free peptide to be washed through the filter. To provide confidence that the free peptide was removed, the VP4N15 peptide alone was processed alongside the VLP-peptide mixture and a VLP-only control. After multiple washes, samples of VLP were retained in the device but the N15 peptide was not detected, suggesting the peptide had been washed away (Figure 2E). In contrast, the peptide-VLP mixture showed bands for both the VLP and peptide. This was consistent with the peptide being in complex with the VLP forming the VLP immunogen, termed HBcPepN15. This material was also visualised by negative stain TEM and particles with similar appearance to the other VLPs were observed (Figure 2C).

### 3.2. Immunisation of Mice with HBcN15 VLPs Induced Both HBcAg- and VP4N15-Specific Immune Responses

Previous work has shown that immunisation of mice with the N-terminal 20 amino acids of EVA71 VP4 displayed on the HBc VLP elicits a neutralising immune response and these antibodies predominantly bound to the N-terminal 15 amino acids [15]. Therefore, immunogens utilising the well-characterised HBc VLP system were used to investigate if an immune response could be generated against the N-terminus of FMDV VP4.

**Figure 3:**
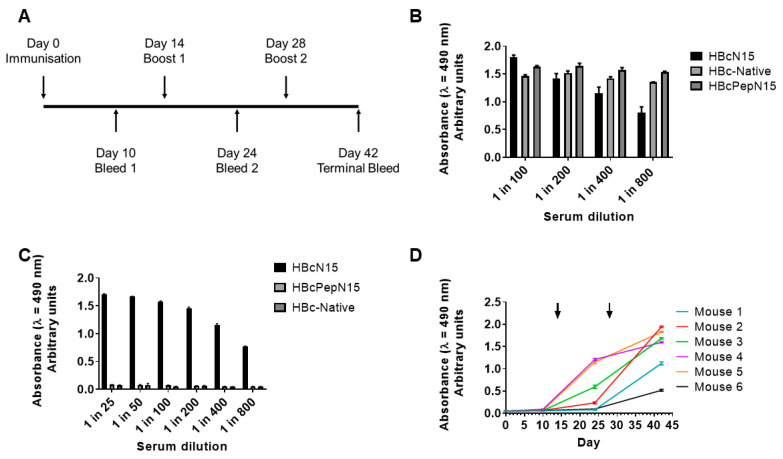
Immune responses to HBcAg VLPs displaying FMDV VP4N15. (**A**) Balb/c mice were immunised with 10 μg of immunogen and boosted over the course of 42 days and test bleeds were collected. ELISAs were used to detect antibodies against (**B**) HBc-native VLPs and (**C**) VP4N15 peptide in pooled terminal sera for each group. The data plotted represent the mean of triplicate wells ± SD and are representative of multiple experiments. (**D**) Peptide ELISAs were used to follow the VP4N15-specific immune response in the HBcN15 group over the course of the immunisations. Arrows indicate booster immunisations.

Groups of mice were immunised three times, at 2-week intervals, with 10 µg of either HBcN15 VLPs, HBcPepN15 VLPs or HBc-native VLPs. Blood samples were collected periodically throughout the experiment (timeline shown in Figure 3A).

Pooled serum from each group was investigated to determine if the immunisations had induced a HBc-native VLP- or VP4N15-specific antibody response. HBc-native VLPs or VP4N15 peptides were used as capture antigens to detect antigen-specific antibodies by ELISA. All three immunisation groups generated an antibody response to the HBcAg scaffold (Figure 3B). However, only the recombinant HBcN15 VLP induced a VP4N15 specific antibody response (Figure 3C).

The immune response in the HBcN15 group was further characterised by looking at the individual responses of each mouse in the group at the different bleeds throughout the experiment (days detailed in Figure 3A). As the experiment progressed, four of the mice began generating VP4N15 antibodies after the first boost, whereas mice 1 and 6 did not start to produce antibodies until after the second boost (Figure 3D). After the second boost, all six mice generated antibodies against VP4N15 (Figure 3D).

Based on the ELISA data presented here, HBcN15 VLPs induced a VP4N15 specific response while HBc PepN15 VLPs did not. As anti-HBcAg antibodies were detected in both groups, this is unlikely to be due to issues with the immunisation and may be due to an issue with the attachment of the peptide to the VLP.

### 3.3. Antibodies against FMDV VP4N15 Are Not Demonstrated to Neutralise FMDV

Previous studies have shown that antibodies against the N-terminus of EVA-71 or HRV VP4 were able to neutralise virus infection and offered some neutralisation against viruses from other serotypes [13,14,15]. The possibility of cross-serotypic antibodies against FMDV is particularly interesting as all serotypes share a completely conserved amino acid sequence at the N-terminus of VP4. Antibodies against the N-terminal 15 amino acids of FMDV VP4 were evaluated for their ability to neutralise a homologous strain of FMDV by plaque reduction neutralisation assay.

FMDV was incubated with either the anti-HBcN15 serum, an existing neutralising bovine polyclonal serum or anti-HBc-native VLP serum and infectious virus titre determined by plaque assay.

**Figure 4:**
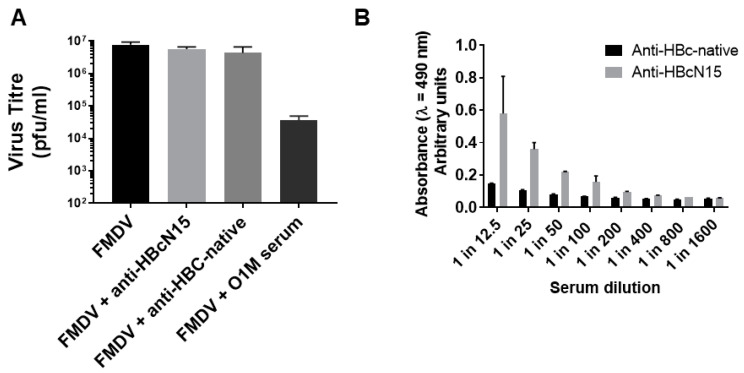
Anti-HBcN15 serum does not neutralise FMDV but does recognise virus particles (**A**) Plaque reduction neutralisation assays were carried out to assess the effect of anti-HBcN15 serum on virus infectivity. Data plotted represent mean of triplicate wells and error bars indicate standard deviation. (**B**) Sandwich ELISAs using bovine αvβ6 integrin as the capture were used to confirm that serum against HBcN15 recognised FMDV virus particles.

After incubation with the neutralising serum against O1M, there was a 2.5 log reduction in virus titre, showing the expected neutralisation of virus infectivity. After incubation with either the anti-HBcN15 or anti-HBC-native VLP serum, there was no change in the virus titre, showing that the neutralisation of FMDV by antibodies against the N-terminal 15 amino acids of VP4 was not demonstrated (Figure 4A).

### 3.4. Antibodies against FMDV VP4N15 Do Recognise Infectious FMDV

The VP4-specific antibodies in the anti-HBcN15 serum were raised against a peptide sequence inserted into the HBc VLP spike rather than against native FMDV. It is, therefore, possible that the VP4 sequence was not adopting a native or virus-like confirmation within the HBc VLP and this would explain why the antibodies generated would not neutralise FMDV. To examine this possibility, the VP4N15-specific serum was tested for its ability to detect FMDV capsids to confirm that the anti-HBcN15 antibodies recognise VP4 in a biologically relevant conformation. 

The virus was captured using recombinant bovine αvβ6 integrin, the natural receptor of FMDV, and the reactivity of the pooled serum from the anti-HBcN15 group was determined. This showed that anti-HBcN15 pooled serum could detect FMDV (Figure 4B), confirming that the antibodies were generated against a biologically relevant confirmation of VP4.

## 4. Discussion

VLPs provide an interesting alternative to whole virus particles for dissecting capsid antigenicity and function and could even be useful tools in controlling economically important diseases such as FMDV. This paper investigated the potential for displaying the highly conserved N-terminus of the VP4 capsid protein of FMDV on a well characterised VLP scaffold, the hepatitis B core. Two approaches were compared for their ability to induce VP4N15-specific antibodies and the antibodies generated were studied.

Previous work by other groups has shown that it is possible to display peptide sequences from the capsids of picornaviruses, such as FMDV VP1 and EVA71 VP4, on the MIR of the HBcAg, and that such modified HBcAg can assemble into VLPs to effectively present the epitope of choice [15,18]. In this study, following a similar design, recombinant VLPs that have the N-terminal 15 amino acids of FMDV VP4 inserted into the MIR have been successfully expressed and purified.

The second approach utilised a previously published technique for decorating a wild-type HBc VLP with tagged peptides using a tag that bound the top of the spike [22]. The VP4N15 peptide was used for this to allow for comparison between the display by epitope insertion and display by peptide attachment.

The two approaches to VP4N15 display were compared in an immunisation study and a VP4N15-specific response was generated against the epitope insertion VLP but not the peptide attachment VLP. The epitope insertion VLP induced VP4N15-specific antibodies in all six mice in the experimental group; however, the level of response did vary between animals.

The peptide attachment group induced a strong immune response against the HBc-native VLP, the scaffold of this immunogen, indicating that the mice had received an adequate dose of the VLPs and that there was not an issue with the administration of the immunogen. As there was no VP4N15-specific response, we conclude that the peptide attachment did not display the epitope as desired.

Although the data in Figure 2 are consistent with the peptide being in complex with the VLP, we present no direct or quantitative evidence of this interaction and it is, therefore, possible that the peptide was not attached or was not attached in the expected orientation. Experiments with additional positive and negative control peptides (e.g., peptide from Blokhina et al. and a peptide without affinity tag, respectively) may have been informative. It is also possible that the 15 amino acid peptide was too short and, when attached to the HBc-native VLP, the orientation and display of the peptide was not sufficient to generate a strong immune response. There is limited structural information about the orientation of the peptide spike binding sequence when bound to the top of the dimer spike, but this suggests that the sequence lies across the dimer and only allows one peptide per spike to bind, hence reducing the risk of steric clashes [21]. There is no information on the predicted orientation of any additional sequence beyond the spike-binding sequence, but from the successful Blokhina et al. study, it seems likely that the sequence is adequately displayed [22]. The peptide here is considerably shorter than that used by Blokhina et al. and may have been too small to be displayed correctly and this could explain the failure to obtain an immune response [22].

Despite the lack of response from the peptide display VLP, the epitope insertion (HBcN15) VLPs did induce a VP4-specific antibody response. The serum from these mice was able to recognise both the VP4N15 peptide and native FMDV particles but did not have a detectable neutralising effect on virus titre. This is somewhat unexpected, as previous work has shown that antibodies against the VP4 sequences of HRV, PV and EVA71 have all been shown to neutralise virus [12,14,15]. In the current study, the method used for the assay of neutralising antibodies was not optimised for sensitivity. It is possible that a more sensitive assay format (e.g., with smaller input titre) would be able to demonstrate some level of neutralisation.

Previous studies with PV have shown that VP4-specific antibodies can bind PV VP4 through the transient externalisation of VP4 at physiological temperatures, during a process known as capsid breathing [12]. Whilst not the aim of the current study, the data showing that VP4N15-specific antibodies can detect FMDV could indicate that capsid breathing is a shared mechanism across picornaviruses of different genera. Alternatively, the relatively weak reactivity of the VP4-specific sera with virus particles and lack of demonstration of neutralising activity could suggest that VP4 is less exposed on the FMDV capsid, relative to PV.

The work presented here has shown that antibodies against the N-terminus of FMDV capsid protein VP4 were successfully generated by peptide display on a recombinant HBc VLP. Although the antibodies did not appear to neutralise FMDV, the HBc VLP system remains an effective option for the future display of epitopes from FMDV and other picornaviruses.

## Data Availability

The authors confirm that the data supporting the findings of this study are available within the article.
